# Women’s empowerment as a determinant of neonatal mortality in Sub-Saharan Africa: a narrative review focused on Nigeria

**DOI:** 10.1080/16549716.2024.2394256

**Published:** 2024-08-28

**Authors:** Joel-Medewase Victor Idowu, Wada Zechariah Ojima, Sayomi Bukola Adetutu, Adetoye Mayowa Mary, Ashaolu Joseph Oluwakayode, Olowolafe Tubosun Alex

**Affiliations:** a Department of Paediatrics and Child Health, Ladoke Akintola University of Technology, Ogbomoso, Nigeria; b Department of Paediatrics and Child Health, LAUTECH Teaching Hospital, Ogbomoso, Nigeria; c Division of Sustainable Development, College of Science and Engineering, Hamad Bin Khalifa University, Qatar Foundation, Doha, Qatar; d Translational Research and Outreach Department, Global Eco-Oasis Sustainable Initiative (GESI), Ibadan, Nigeria; e Department of Public Health, Lead City University, Ibadan, Nigeria

**Keywords:** Neonates, sustainable development goal 3, women’s empowerment, gender equality, women and girls

## Abstract

Neonatal mortality remains a critical public health issue, with Sub-Saharan Africa (SSA) experiencing disproportionately high rates compared to other global regions. Notably, SSA and South Asia are the regions most lagging behind the Sustainable Development Goal (SDG) 3.2, aiming for <12 neonatal deaths per 1,000 live births by 2030. Within SSA, Nigeria, the most populous country, records the highest number of neonatal deaths annually. Given the structural similarities among SSA nations, this narrative review, focusing on Nigeria, explores effective strategies to reduce the neonatal mortality gap. Information about trends, risk factors, and prevalent lapses was obtained from literature from renowned databases like PubMed, Scopus, and Google Scholar, and grey literature consisting of reports from relevant governmental and non-governmental organizations. Critical risk factors commonly identified include inadequate antenatal care (less than three visits), lack of access to skilled and clean birth practices, limited healthcare accessibility, financial barriers, substandard environmental conditions, and nutritional shortfalls. This review highlights women’s empowerment as an additional critical factor, often overlooked, in the efforts to decrease neonatal mortality rates. Improving women’s empowerment indices, such as the Gender Inequality Index (GII), employment, and literacy, offers a promising avenue to curtail neonatal mortality rates in Nigeria and across SSA sustainably. While this is potentially a long-term solution, short and medium-term recommendations were also proffered. By integrating women’s empowerment within a broader strategy to improve maternal and newborn health, Nigeria can advance towards securing a healthier future for its youngest population.

## Paper Context

- **Main findings:** Neonatal mortality remains a major public health crisis in SSA, even with sustained local and global efforts.
- **Added knowledge:** Sustainably resolving this crisis requires a holistic approach that includes women’s empowerment, a factor that is often overlooked in current interventions to curb neonatal mortality.
- **Global health impact for policy and action:** Prioritizing women’s empowerment will contribute to sustainably reducing neonatal mortality rates and will also help address other prevalent public health and economic challenges facing developing countries.

## Background

The neonatal period, spanning the first 28 days of life, represents the most critical phase for a newborn’s survival. During this time, the risk of mortality peaks, with studies indicating that more than 80% of neonatal deaths occur within the initial week [1]. In 2022 alone, the global neonatal mortality rate stood at 17 deaths per 1,000 live births, culminating in approximately 2.3 million neonatal deaths worldwide, or about 6,300 each day [2]. Reducing neonatal mortality rates is crucial for lowering the overall under-5 mortality rate; notably, in 2015, neonatal deaths accounted for 46% of all under-5 fatalities [3]. A population-based multi-country study examining neonatal mortality risk among 125.5 million births from 15 countries revealed that preterm birth was the most significant risk factor for neonatal mortality [4]. Other commonly reported factors that leave neonates susceptible are sepsis, low birth weight, and respiratory distress syndrome [5]. Conversely, interventions, like increased antenatal clinic attendance (more than three visits) and community health worker home visits, have been shown to reduce the likelihood of neonatal death substantially [6].

Despite the global commitment to reduce child mortality, as outlined in Sustainable Development Goal (SDG) 3.2—which aims for fewer than 12 neonatal deaths per 1,000 live births by 2030 [7]—Sub-Saharan Africa (SSA) remains significantly behind this target. In contrast to several countries worldwide—such as Albania, Argentina, Australia, Austria, Brazil, Cyprus, Jordan, Norway, Oman, Peru, Sri Lanka, Thailand, the United Kingdom, and the United States—which reported neonatal mortality rates below 10 per 1,000 live births in 2021, many SSA nations, including Zimbabwe, Togo, Sierra Leone, Senegal, Niger, Nigeria, Mali, and Madagascar, recorded rates exceeding 20 per 1,000 [8,9]. Notably, South Sudan, Pakistan, Somalia, Nigeria and Lesotho are the countries with the highest neonatal mortality rates globally, with over 34 per 1,000 [10]. This discrepancy highlights a significant challenge, as, apart from South Asia—with 23 deaths per 1,000 live births—SSA, with a rate of 27 per 1,000, stands as the only region not meeting or surpassing the SDG 3.2 target [8], underscoring the urgent need for focused interventions and strategies to address this critical issue in these regions.

Thus, this mini-narrative review critically examines the recent trends in neonatal mortality within Nigeria, Africa’s most populous country, and identifies key factors contributing to this pressing public health issue. Given Nigeria’s significant share of neonatal deaths in SSA and its representation of wider regional challenges, our investigation is not only timely but essential. While previous studies have laid the groundwork in this field, there is a continuous need for up-to-date analysis to unravel the complex web of factors leading to high neonatal mortality rates. This study seeks to bridge that gap, offering insights into the nuances of neonatal mortality trends across Nigeria and evaluating associated risk factors. By doing so, it aims to craft actionable recommendations that could significantly impact policy formation and implementation. In light of SDG 3.2, which targets a substantial reduction in global neonatal mortality by 2030, our review is poised to contribute meaningfully towards achieving this objective. Through this analysis, we aspire to spark a discourse that will strengthen efforts towards safeguarding neonatal lives in Nigeria and, by extension, SSA, thereby inching closer to meeting the global mandate of reduced child mortality.

## Methodology

This narrative review focused on literature published from 2015 to the present. Papers exclusively using pre-2015 data were excluded. This timeframe was chosen to align with monitoring trends and performance since the inception of the SDGs as the target period approaches its final five years. The review employed a systematic approach to identify relevant studies, utilizing specific keyword combinations such as ‘Neonate’ and ‘Nigeria’, ‘Neonate and sub-Saharan Africa’, ‘Neonatal mortality and Nigeria’, ‘Neonatal fatality and Nigeria’ to assess neonatal mortality in Nigeria. In tandem, keywords like ‘Gender inequality index’, ‘Women’s literacy rate’, and ‘Women’s labour participation’ were used to find reports to correlate women’s empowerment with neonatal mortality rate. The keywords were cross-referenced with the article title, abstract, and keywords from the relevant databases.

The search was conducted across multiple databases, including Google Scholar, PubMed, and Scopus. In addition to peer-reviewed journal articles, grey literature was incorporated to ensure a comprehensive overview. This included reports from reputable organizations such as the World Bank Group, United Nations International Children’s Emergency Fund (UNICEF), United Nations Development Programme (UNDP), African Development Bank, and the Nigeria Demographic and Health Survey (NDHS), providing the most recent data at national and regional levels.

Descriptive statistics were provided in frequencies and proportions for neonatal mortality and women’s empowerment variables. These were represented in charts and figures. Additionally, inferential statistical analyses were performed at a 95% confidence interval to ascertain statistically significant associations between neonatal mortality rates and women’s empowerment variables like women’s literacy rate, gender inequality index, and out-of-school-girls rate across Nigeria’s six geopolitical zones and Africa. Pearson correlations were run to determine the level of association between both variables and the strength of the association using the Pearson *R* value. *R* values between 0.5 and 1 indicated a strong correlation between both variables, values between 0.3 and 0.49 indicated a moderate correlation, values less than 0.3 indicated a low correlation, while zero indicated there was no correlation. Assumption checks were performed before the analysis to ensure compatibility with the test method. In instances where the Shapiro-Wilk’s test was statistically significant (p < 0.05), indicating non-normality, Spearman’s rho correlation was used in place of Pearson’s R. Furthermore, the Fisher’s Z effect size was used to complement the *R* values when the sample size was small. This approach is important because, with small sample sizes, the variance of Pearson’s coefficient increases, decreasing the precision of its estimates. Using Fisher’s Z helps stabilize the variance and obtain more precise estimates of the correlation strength within small samples.

## Trends of neonatal mortality in Nigeria and across Africa

**Figure 1.**
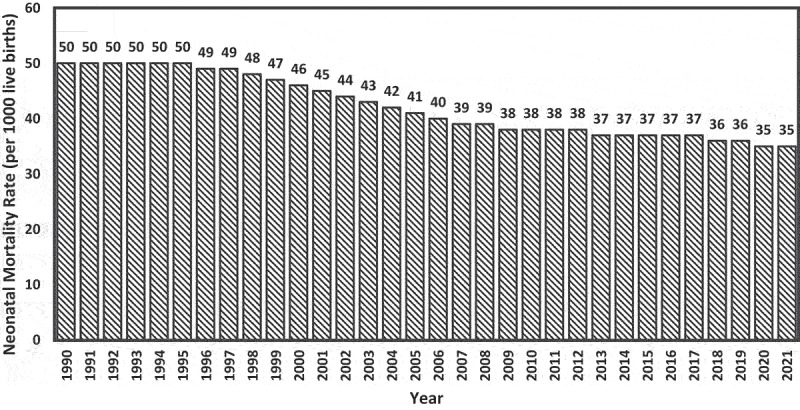
Trends of neonatal mortality rate in Nigeria (1990 to 2021).

According to a UNICEF report, in Nigeria, about 700 babies die every day before completing four weeks, while around 840 stillbirths occur daily [11,12]. Nigeria’s neonatal mortality rate has only slightly declined over the past three decades, from 50 in 1000 live births in 1990 to 35 in 1000 live births in 2021 [8]. This figure is still higher than the average mortality rate of 27 in 1000 for SSA and is the second highest in the world. Nigeria ranks as the second highest globally in annual neonatal deaths, trailing only behind India, and is recognized as having the second-largest number of maternal and child deaths worldwide [13,14]. Figure 1 highlights the trends of neonatal mortality rates in Nigeria from 1990 to 2021, according to the World Bank data [8].

In Nigeria, prematurity, birth asphyxia, and low birth weight are critical factors in neonatal mortality, with the risk of death rising sharply as birth weight decreases [12,15,16]. A significant number of neonates with low birth weight are born preterm, rendering them vulnerable to prematurity-associated complications such as asphyxia, apnea, anaemia, and infections, all of which substantially increase the risk of mortality [1,17,18]. This is highlighted in at least 70% of the studies examined in this review, as shown in Table 1. Neonates born at term but with low birth weight often suffer from intrauterine growth restriction or have concurrent conditions that impair their prenatal development [38,39], and they are also more prone to life-threatening congenital anomalies, further jeopardizing their chances of survival [4]. Preterm delivery is linked to factors such as low socio-economic status, a history of preterm delivery, antepartum haemorrhage, premature rupture of membranes, pregnancy-induced hypertension, and the mother’s booking status at the healthcare facility [40]. To prevent preterm delivery, healthcare service providers should be able to identify pregnant women at risk, treat infections, and counsel on nutrition and micronutrient supplementation in pregnancy [41].

**Table 1.** Literature on Neonatal Mortality from Hospital-based Studies in Nigeria from 2015 till May 2024.

Region	Neonatal Mortality Rate	State	Year	Area (Urban/Rural)	Leading Causes	References
North West						
	71.6/1,000	Jigawa	2018	Birnin Kudu (Rural)	Birth asphyxia, prematurity, and jaundice	[19]
	200.4/1,000	Zamfara	2017	Gusau (Urban)	Birth asphyxia, prematurity, and neonatal sepsis	[20]
	132/1,000	Jigawa	2020	Dutse (Urban)	Prematurity, neonatal sepsis, and severe birth asphyxia	[21]
	66/1,000	Zamfara	2021	Gusau (Urban)	Birth asphyxia, prematurity, and neonatal sepsis	[22]
North East						
	–	–	–	–	–	–
North Central						
	135.8/1,000	Jos	2020	Plateau (Urban)	Prematurity, birth asphyxia, and neonatal sepsis	[23]
	114/1,000 (inborn), 178/1,000 (outborn)	FCT	2021	Abuja (Urban)	Prematurity, congenital malformations, and perinatal asphyxia	[24]
	105/1,000	Nasarawa	2021	Lafia (Urban)	Asphyxia, respiratory distress syndrome, and neonatal sepsis	[25]
	138/1,000	FCT	2020	Abuja (Urban)	-	[26]
	87-121/1,000 (Term babies), 173-202/1,000 (Preterm babies)	Kwara	2022	Ilorin (Urban)	Severe perinatal asphyxia, bilirubin encephalopathy, respiratory distress, and congenital malformation (Term babies)Severe perinatal asphyxia, sepsis, respiratory distress, acute bilirubin encephalopathy, intraventricular haemorrhage, and congenital malformation (Preterm babies)	[27]
South West						
	27.2/1,000	Ogun	2019	Shagamu (Semi-urban)	Severe perinatal asphyxia and prematurity	[28]
	28.4/1,000	Lagos	2017	Makoko and Badia East (Rural)	Delivery in non-hospital settings	[29]
	129/1,000 (inborn), 223/1,000 (outborn)	Ekiti	2021	Ado-Ekiti (Urban)	Low birth weight, birth asphyxia, prematurity, neonatal sepsis, and respiratory distress syndrome.Delivery in non-hospital settings	[30]
	103/1,000	Oyo	2019	Ogbomosho (Semi-urban)	Prematurity and perinatal asphyxia	[31]
	146/1,000 (among low birth weight)	Ondo	2022	Akure (Urban)	Low birth weight and neonatal sepsis	[32]
	125/1,000	Lagos	2019	Lagos (Urban)	Asphyxia, jaundice, sepsis, and prematurity	[33]
South East						
	60/1,000	Imo	2019	Owerri (Urban)	Perinatal asphyxia, prematurity, and neonatal sepsis	[34]
	226/1,000	Imo	2024	Orlu (Urban)	Prematurity, perinatal asphyxia, and neonatal sepsis	[35]
South South						
	12/1,000 (antenatal care), 31/1,000 (without antenatal care)	Edo	2022	Benin City (Urban)	Antenatal care with the hospital of delivery	[36]
	60/1,000	Cross River	2020	Calabar (Urban)	Prematurity, perinatal asphyxia, neonatal sepsis, and congenital malformation	[37]

**Figure 2.**
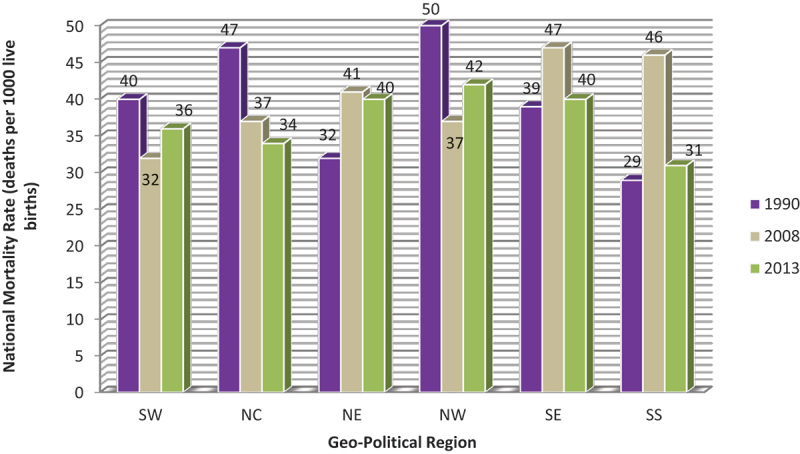
Trends in neonatal mortality rate in Nigeria geo-political regions, 1990–2013 [23,26,28].

Akinyemi et al. [42]. examined the trend of neonatal mortality rates in Nigeria from 1990 and attributed high fatality rates to factors like inadequate antenatal care, short birth intervals, delivery in non-formal settings, and small birth size. Similar factors have been reported in other studies [43,44]. While examining the trends in Figure 2 closely, there was a noticeable increase in the neonatal mortality rate in 2008, which could be attributed to the significant rise in the Nigerian rural population [45], which typically lacks access to standard healthcare services [46]. Between 1990 and 2003, the rural population remained around 65%, while the urban population was about 35% [42]. However, by 2008, the rural population had increased to 73.5% [45]. By 2013, the rural population had decreased to 67% [47], corresponding with a decrease in neonatal mortality rates in most regions, as shown in the trend. Additionally, the prevalence of home deliveries increased to 67.3% in 2008, compared to 60% in 1990 and 63.9% in 2013 [42,45,47]. Home deliveries are known risk factors for neonatal mortality due to the limited accessibility to necessary healthcare services in the event of complications [1,48]. These factors combined could explain the rise in neonatal mortality rates in 2008 and the overall small differences or increases in neonatal mortality rates between 1990 and 2013.

**Figure 3.**
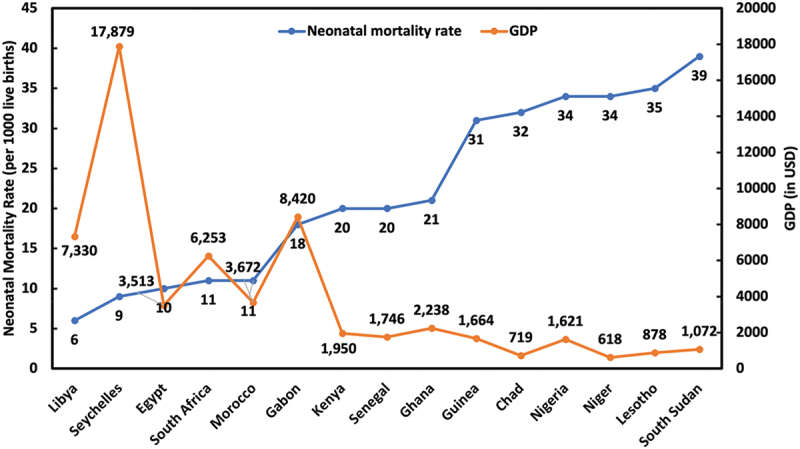
Correlation between GDP per capita and neonatal mortality rate across selected African countries (*p* < 0.01; *R* = −0.867; Fisher’s z = −1.1319).

Despite improvements across the country, the northwest and northeast regions report the highest rates of neonatal mortality (Figure 2), whereas the southeast and southwest regions have the lowest [42]. This is most probably because the Northeast and Northwest regions are the poorest in Nigeria and most deprived of education, living conditions, and health [49]. Similarly, on a continental scale within Africa, economic factors are correlated with neonatal mortality rates (Figure 3). For example, countries such as Seychelles, South Africa, Egypt, Morocco, and Libya, which have gross domestic products (GDP) per capita over 3,500 USD [50], achieve neonatal mortality rates within the SDG 3.2 target [8]. Conversely, countries with some of the lowest GDPs, including South Sudan, Lesotho, Niger, Chad, and Nigeria, exhibit some of the highest neonatal mortality rates on the continent, ranging from 32 to 39 deaths per 1,000 live births [8,50]. Statistical analysis also affirmed a statistically significant strong negative correlation between GDP and neonatal mortality rate (p < 0.01; *R* = −0.867; Fisher’s z = −1.1319).

However, exceptions like Gabon illustrate that economic factors alone do not fully explain neonatal mortality rates. Despite Gabon’s high GDP of 8,420 USD [50], the country has a neonatal mortality rate of 18 [8], largely due to severe economic inequality. Although Gabon is rich in natural resources such as oil and minerals, about 40% of its population lives below the global poverty threshold [51]. Moreover, as of 2022, half of the population lacked access to clean water, and while 90% of urban residents had access to electricity, 70% of rural inhabitants did not [51]. Similarly, even though Nigeria has a GDP per capita higher than Chad and similar to Guinea and Senegal, it has a higher neonatal mortality rate, which is most probably attributed to having the largest population in the continent and the highest number of people (around 80,000,000) living below the poverty threshold [52].

This demonstrates that while economic indicators are vital for addressing neonatal mortality and other public health challenges, solutions must be tailored to local contexts and specific needs. Hence, although increased GDP per capita is expected to reduce neonatal mortality rates in most sub-Saharan African countries, nations like Gabon require targeted interventions to address local social and economic disparities.

Neonatal deaths are most frequent on the first day of life, with approximately 45% occurring within the first 24 hours and 75% within the first week [53]. This pattern suggests the critical nature of the immediate postnatal period, as survival beyond the first week indicates a successful adaptation to life outside the womb [54]. Moreover, infants born before 28 weeks of gestation face a significantly higher risk of dying during the neonatal period, with preterm babies being twelve times more likely to encounter morbidity and mortality than term babies [55]. Mokuolu et al. [40]. reported a mortality rate as high as 64% in babies born at less than 28 weeks, likely due to their lower cortisol levels, which are essential for postnatal adaptation [54]. The mortality rate for preterm babies is expected to be higher in non-hospital settings, as intensive care is vital for their survival. According to a recent report by the WHO, over 90% of preterm babies born before 28 weeks in low-income countries die within the first few days of life, compared to only 10% in high-income countries [56]. Table 1 provides results from hospital-based neonatal mortality studies from 2015 to June 2024. From the table, it can be seen that the most prevalent morbidities predisposing neonates to mortality in Nigeria are prematurity, sepsis, and perinatal asphyxia.

## Risk factors of neonatal mortality in Nigeria

Neonatal mortality in Nigeria predominantly stems from causes that are both preventable and treatable [57–61]. In this section, the most prevalent risk factors contributing to the high rates of neonatal deaths across the country are examined. This section uncovers the economic, social, physical, and cultural barriers that significantly impact neonatal survival by analyzing recent studies. The comprehensive understanding of the risk factors will not only shed light on the complexities of neonatal mortality in Nigeria but also enable us to propose adequate recommendations to mitigate these risks and ultimately reduce the number of neonatal deaths.

Recent strategies to improve pregnancy outcomes in Nigeria have significantly focused on enhancing the quality of antenatal care and ensuring that skilled birth attendants are present during deliveries [62–64]. While it is difficult to conclusively determine if the deployment of skilled birth attendants has significantly curbed neonatal mortality rates in recent years, recent literature suggests that women of reproductive age, particularly in rural and conflict-affected areas, continue to widely support the use of both skilled and traditional birth attendants [65,66]. Some reasons for this preference include the accessibility of the centres, affordability of services, warm and hospitable staff, and perceived spiritual support [65–68]. However, numerous studies have confirmed that deliveries outside formal healthcare settings are still associated with higher rates of neonatal mortality and morbidity [1,69], indicating that lapses still exist. Despite these efforts, progress in reducing neonatal mortality rates has been minimal over the last decade, with a reduction from 37 to 35 deaths per 1,000 live births. This stagnation is attributed to several factors, including inadequate access to quality neonatal healthcare, the prevalence of non-hospital, unhygienic birth settings [44,64], and out-of-pocket healthcare payments that limit access to quality care [44,60]. Environmental conditions, such as lack of clean/potable water and overcrowding, along with financial hardship, continue to exacerbate the neonatal mortality rate [58,64,70–73]. Additionally, location has also been found to play a major role in the outcome of pregnancies in Nigeria [62,74]. Mundi et al. and Ojima et al. [1,58] reported lower incidences of neonatal mortality in urban areas, where pregnant women have access to specialized care and better hospital facilities, including neonatal services, compared to rural areas.

While these factors are critical, the empowerment of women emerges as an additional, yet often underexplored, element in the effort to reduce neonatal mortality rates. Empowering women can significantly enhance their capacity to access basic healthcare services, improve environmental and living conditions, and overcome financial hardships that limit neonatal survival. Thus, the next section of this review explores how improving women’s empowerment indicators could contribute significantly to the collective efforts needed to meet the SDG 3.2 target by 2030.

## Women’s empowerment: a critical component in sustainably reducing neonatal mortality rates in Nigeria and Sub-Saharan Africa

The socioeconomic status of women in the reproductive age group significantly impacts neonatal outcomes in Nigeria. Education level, a key component of socioeconomic status, plays a pivotal role in the health and survival of newborns [64,75]. Lack of quality education limits a woman’s decision-making capacity, increases dependency on spouses, and restricts access to healthcare services, leading to delayed or no emergency interventions [74]. Fagbeminiyi et al. [76] highlight this ‘dependency syndrome’ as a direct contributor to increased neonatal mortality rates.

Research by Aderanti et al. [77] and Nwabueze et al. [62] underscores the strong correlation between a mother’s educational attainment and her awareness of neonatal survival risk factors. Women with higher education levels demonstrate better neonatal survival rates than their less educated counterparts. This pattern is echoed in findings across various studies, including the Nigeria Demographic and Health Survey (2013), which noted higher under-5 mortality rates among children of mothers with primary or secondary education levels compared to those whose mothers attained tertiary education [47].

The ability of women to generate income, closely tied to their educational background, also plays a crucial role in influencing health and nutritional choices during pregnancy [70]. Adequate nutrition is essential for maintaining healthy maternal and fetal tissues, with poor maternal nutrition linked to increased maternal morbidity and neonatal mortality rates. The provision of quality healthcare and nutritional support is very vital for all women, especially pregnant women. Good nutrition is essential for physical growth, mental development, performance, productivity, health, and well-being across an entire lifespan, making nutrition a sound investment for any country [70,78]. Maternal malnutrition is particularly worse in low-income countries like Nigeria, where undernutrition, inadequate food intake, and micronutrient deficiencies exist [70].

Further evidence of the association between women’s empowerment indicators and neonatal mortality rates can be observed regionally across Nigeria (Table 2). According to the 2018 NDHS data, the Northwestern and Northeastern Nigeria geopolitical zones had the highest neonatal mortality rates, 46 and 37 deaths per 1,000 live births, respectively, as opposed to other regions with rates of less than 33 deaths per 1,000 live births [79]. When comparing the differences between these regions based on women’s empowerment indicators, it was discovered that the neonatal mortality rate was directly proportional to women’s literacy rate, out-of-school girls’ rate for both primary and secondary schools, and women’s employment rate [79,80]. For example, women’s literacy was as low as 29% and 32% in Northwestern and Northeastern Nigeria, while the values ranged from 50% to 81% across the other regions [79]. In addition, when considering other factors like the accessibility of women to the internet and mass media, only 7.2% and 10.4% of women in Northwestern and Northeastern Nigeria have had access to the internet at a point, while only between 38% and 50% had been opportune to be exposed to mass media at least once a week [80]. These imply that women in these regions will most probably be unaware of the health promotion campaigns on the internet and in the mass media (radio, television, and newspapers). Results from inferential statistics affirmed there were statistically significant strong correlations between neonatal mortality rate and literacy rate (p = 0.05; *R* = −0.812; Fisher’s z = −1.132), out-of-school-girls (p = 0.008; R = 0.928; Fisher’s z = 1.641), women’s employment rate (p = 0.05; *R* = −0.812; Fisher’s z = −1.132) and internet usage among women (p = 0.05; *R* = −0.812; Fisher’s z = −1.132). Overall, the data shows that the socio-economic disparities between women in northern and southern Nigeria are most probably significant contributors to the disproportional neonatal mortality rates. On the other hand, non-socioeconomic variables, like female genital mutilation rate and gender-based violence, did not seem to have any direct impact on neonatal mortality rates as these values were significantly higher in southern Nigeria as opposed to northern Nigeria.

**Table 2.** Correlation between women’s empowerment variables and neonatal mortality rate across the geopolitical regions in Nigeria.

Geopolitical Zones	North West	North East	North Central	South West	South East	South South	References
Women Literacy (%)* (*p* = 0.05; *R* = −0.812; Fisher’s z = −1.132)	29.0	31.8	49.6	80.6	79.3	79.0	[60]
Out-of-school girls for primary school age (%)* (*p* = 0.008; *R* = 0.928; Fisher’s z = 1.641)	29.8	42.4	24.8	14.3	11.1	13.1	[61]
Out-of-school girls for secondary school age (%)* (*p* = 0.008; *R* = 0.928; Fisher’s z = 1.641)	36.8	38.1	22.3	11.2	7.8	8.4	[61]
Women employment rate* (*p* = 0.05; *R* = −0.812; Fisher’s z = −1.132)	50.8	60.7	70.9	78.1	71.8	72.7	[60]
Women’s exposure to mass media at least once weekly (%) (*p* = 0.173; *R* = −0.638; Fisher’s z = −0.754)	50.2	37.9	43.4	80.7	65.1	72.7	[61]
Women that have ever used the internet (%)* (*p* = 0.05; *R* = −0.812; Fisher’s z = −1.132)	7.2	10.4	18.5	50.6	36.1	36.7	[61]
Reproductive rights (FGM) (*p* = 0.354; *R* = −0.464; Fisher’s z = −0.502)	20.2	6.1	9.9	30.0	35.0	17.7	[60]
Gender-based violence (*p* = 0.577; *R* = −0.290; Fisher’s z = −0.298)	38.3	11.7	43.3	29.8	36.1	46.4	[60]
Neonatal mortality rate	46 per 1000	37 per 1000	32 per 1000	31 per 1000	27 per 1000	27 per 1000	[60]

*Represents variables that had statistically significant associations and strong correlations with neonatal mortality rate.

**Figure 4.**
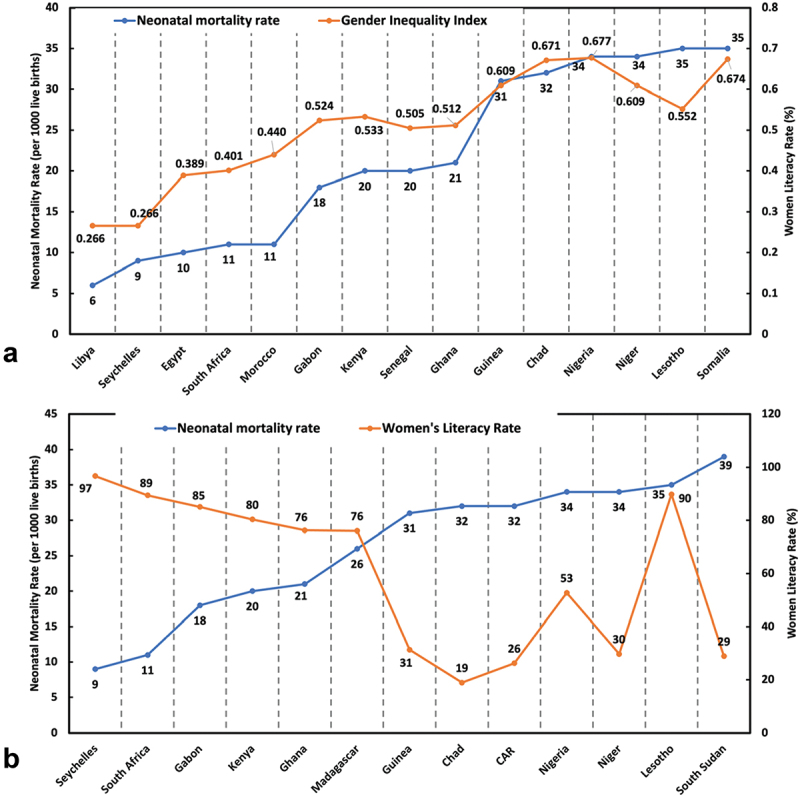
(a): correlation between neonatal mortality rate and GII across selected African countries (*p* < 0.001; *R* = 0.915; Fisher’s z = 1.556); (b) correlation between neonatal mortality rate and women’s literacy rate across selected African countries (*p* = 0.004; *R* = −0.740; Fisher’s z = −0.950).

Further evidence that women’s empowerment significantly reduces neonatal mortality rates and other related maternal well-being targets can be seen by examining the Gender Inequality Index (GII) across African countries (Figure 4a). There was a statistically significant association, with a strong positive correlation between both variables (*p* < 0.001; *R* = 0.915; Fisher’s z = 1.556). The UNDP defines GII as a composite metric for gender inequality using three dimensions: reproductive health, empowerment, and labour market participation [81]. The GII value ranges from 0 to 1, with lower values indicating low inequality between women and men [82]. African countries like Seychelles (rank 67), Libya (2), South Africa (110), and Egypt (105) have the lowest neonatal mortality rates and meet the SDG 3.2 target [81]. These countries are categorized as having high human development with GII values between 0.266 and 0.401, and their global ranks are between 67^th^ and 110^th^ among the 193 countries examined in the UNDP report [81]. Conversely, countries with the highest neonatal mortality rates in Africa, such as Chad (189), Nigeria (161), Niger (189), Lesotho (168), and Somalia (193), are categorized as having low human development and have GII values between 0.552 and 0.677, with global ranks between 161^st^ and 193^rd^ [81]. These findings clearly demonstrate that higher gender equality, as measured by the GII, correlates with lower neonatal mortality rates. Countries with better gender equality and higher human development indices tend to have more favourable neonatal outcomes, underscoring the importance of empowering women to improve public health metrics.

The correlation between women’s empowerment and the neonatal mortality rate is further evident when examining women’s literacy data across Africa from the World Bank Group [83]. A statistically significant strong negative correlation (p = 0.004; *R* = −0.740; Fisher’s z= −0.950) was observed between women’s literacy and neonatal mortality rates (Figure 4b). Countries with low neonatal mortality rates, such as Seychelles and South Africa, have high women’s literacy rates of 96.7% and 89.4%, respectively [8,83]. In contrast, countries with neonatal mortality rates exceeding 30 deaths per 1,000 live births generally have women’s literacy rates below 35%. For instance, South Sudan and Niger, where the literacy rate is under 30%, have neonatal mortality rates of 34 and 39 deaths per 1,000 live births, respectively [8,83]. Notable exceptions include Nigeria and Lesotho, with women’s literacy rates of 52.7% and 89.8%, respectively, yet both countries still experience neonatal mortality rates of about 35 deaths per 1,000 live births. In Nigeria, this discrepancy can be attributed to the large population size, resulting in the highest number of people living below the global poverty threshold [52], as previously mentioned. In Lesotho, despite a high literacy rate among women, the country’s low GDP per capita (878 USD), significant poverty levels, and high inequality rates contribute to its high neonatal mortality rate [50,84].

Besides education and socioeconomic status, cultural practices also affect women’s well-being and, consequently, their newborn infants. For example, during the Echeroko festival of Eku in Southern Nigeria, women are not allowed to go out at certain durations, as doing so will attract punishment from the gods, notwithstanding her clinical condition, even when she is pregnant [85]. Also, in the northern part of the country, where the purdah system is practised, the women’s movement is restricted as this aims to prevent them from being seen by other men [86]. Worse still, medical practitioners are not allowed during delivery [87]. The culture of the Yoruba people also enforces restrictions on any form of access for women during traditional rites, even in medical emergencies. For example, during the Oro festival, there is no freedom of movement for women, as they are forbidden to go out in the open, where they might encounter Oro deity worshippers on the way [1,71]. Similarly, some cultures do not permit the immunization of newborns, which helps prevent severe diseases in the infant [88].

Overall, these findings clearly demonstrate that higher gender equality, as measured by the GII, and a higher literacy rate among women correlate with lower neonatal mortality rates. These challenges highlight the urgent need for initiatives and interventions to empower girls and women. A notable reduction in the neonatal mortality rate is anticipated by prioritizing and enhancing their education and socioeconomic status. Education not only equips women with the knowledge to make informed health decisions but also improves their access to healthcare services, enhancing maternal and neonatal outcomes. Furthermore, empowering women economically enables them to make better nutritional choices for themselves and their offspring, addressing one of the critical pathways to reducing neonatal mortality rates. Addressing cultural practices that negatively impact women’s and newborns’ health is equally crucial. Nigeria and SSA can make significant strides towards achieving the SDG 3.2 target within the next decade by fostering a society that values and supports women’s health and rights.

## Conclusion

Addressing the multifaceted challenge of the high neonatal mortality rate in Nigeria requires a holistic approach, with women’s empowerment as one of the key criteria to consider. Enhancing women’s socioeconomic status will improve access to quality healthcare and nutrition and overcome cultural barriers, which are critical factors influencing neonatal mortality rates. This long-term strategy will yield significant benefits over time. Monitoring and improving indices such as the Gender Inequality Index (GII), women’s literacy, out-of-school girls’ rate, and women’s labour participation are crucial for economic progress and enhancing public health outcomes.

In the short term, skilling up traditional birth attendants (TBAs) and integrating them into the formal health system is a practical solution, especially in rural and remote areas. TBAs are trusted in these regions, and their inclusion can bridge gaps in neonatal care at a low cost. Registering TBAs with health boards, monitoring their output, and ensuring regular training and license renewal are essential. In the medium term, strengthening the referral system, improving road networks, and providing equipped transportation for referrals to secondary or tertiary facilities are vital. Equipping these centres with necessary medical equipment and skilled personnel will further support advanced newborn care.

Overall, this review recommends integrating women’s empowerment into the broader strategy for enhancing maternal and newborn health, enabling Nigeria and similar countries to make significant strides towards creating a healthier future for its youngest citizens.
